# Lipin‐1 determines lung cancer cell survival and chemotherapy sensitivity by regulation of endoplasmic reticulum homeostasis and autophagy

**DOI:** 10.1002/cam4.1483

**Published:** 2018-04-16

**Authors:** Xueyu Fan, Yuanyuan Weng, Yongfeng Bai, Zongpan Wang, Siwei Wang, Jin Zhu, Feng Zhang

**Affiliations:** ^1^ Core Facility Department of Clinical Laboratory Quzhou People's Hospital Quzhou, Zhejiang China; ^2^ Department of Oncology Quzhou People's Hospital Quzhou, Zhejiang China; ^3^ Department of Pharmacology Quzhou People's Hospital Quzhou, Zhejiang China

**Keywords:** Autophagy, cisplatin, endoplasmic reticulum stress, lipin‐1, lung adenocarcinoma

## Abstract

Cancer cells undergo comprehensive metabolic reprogramming to meet the increased requirements of energy and building blocks for proliferation. Lipin‐1, a phosphatidic acid phosphatase converting phosphatidic acid (PA) to diacylglycerol (DAG), is upregulated in lung adenocarcinoma (LUAD) cell lines and tumor tissues. In this study, we reveal high lipin‐1 expression is correlated with poor prognosis of patients with LUAD. Knockdown of lipin‐1 decreases cell viability and proliferation in LUAD cells, whereas it has less effect on nontumorigenic lung cells. Autophagy and ER stress play important roles in tumor initiation and progression. Lipin‐1 knockdown induces the initiation of autophagy while disrupts formation of autolysosome. Lipin‐1 silencing induces the activation of ER stress through the IRE1*α* pathway. Furthermore, we demonstrate disrupted ER homeostasis contributes to the cell phenotype, and the elevated autophagy initiation is due to the ER stress in part. For the first time, we show lack of lipin‐1 enhances the sensitivity of LUAD cells to cisplatin treatment. Our results suggest that lipin‐1 is a potential target, alone or combined with other treatment, for lung cancer therapy.

## Introduction

Cancer cell undergoes comprehensive metabolic reprogramming to meet the increased requirements of energy and building blocks for proliferation. Phospholipids are the crucial material not only required for cell membrane construction but also play significant roles as signaling molecules. Nevertheless, the alteration of phospholipid metabolism in tumor initiation and progression is unclear. As a metabolic enzyme for phosphatidic acid, lipin‐1 is a unique bifunctional protein. Lipin‐1 is able to catalyze phosphatidic acid (PA) to diacylglycerol (DAG) in the process of triglycerides and phospholipid biosynthesis. It also serves as a coregulatory factor for transcription, which can upregulate genes related to fatty acid uptake and oxidation 1, 2. Due to its dual function as an enzyme and a transcriptional regulator, lipin‐1 plays a special role in regulating intracellular lipid metabolism. So far, lipin‐1 is widely reported to be crucial to the formation of adipose tissue in vivo 1, 3. However, the effect of lipin‐1 in tumor pathology is largely unknown. Very recently, lipin‐1 was reported to be required for prostate and breast cancer cell survival. Knockdown of lipin‐1 by siRNA decreases prostate cancer cell proliferation and migration 4.Overexpression of lipin‐1 correlates with the poor prognosis of patients with triple‐negative breast cancer (TNBC). Deficiency of lipin‐1 induces apoptosis in basal‐like TNBC cell lines 5.

During the response to various stresses, cells undergo rapid changes to protect themselves against potential damage. This is orchestrated through a multifaceted cellular program, which involves diverse stress response pathways. Autophagy and unfolded protein response (UPR) are the key pathways that mediate stress‐induced damage control. Autophagy is a highly regulated cellular process by which cells remove damaged proteins and organelles from themselves to survive starvation and stress. In normal tissues, autophagy‐mediated damage mitigation may suppress tumorigenesis, while the role of autophagy in cancer is complicated. Autophagy acts to either promote or inhibit tumorigenesis dependent on tumor type, stage, and genetic context. Currently, a variety of commonly used chemotherapeutic agents induce autophagy directly or indirectly. Autophagy has been widely considered as a critical regulator of multiple aspects of cancer pathology. In fact, autophagy can be integrated with other cellular stress responses through either parallel stimulation of autophagy and other stress responses by specific stress stimulator through mutual control of autophagy and other stress responses.

Disturbances in the normal functions of the endoplasmic reticulum (ER) lead to ER stress, an evolutionarily conserved cell stress response. Upon ER stress, cells activate a series of adaptive mechanisms to compensating for damage, which together are known as UPR 6. Initially, the UPR transduces information about ER stress to the nucleus and cytosol to buffer fluctuations in unfolded protein load. When ER dysfunction is severe or prolonged, this pathway triggers cell death to eliminate damaged cells 7. Interestingly, it has been reported that autophagy is activated for cell survival after ER stress 8.

In this study, we showed that lipin‐1 is significantly upregulated in patients with lung adenocarcinoma and overexpression of lipin‐1 is highly correlated with poor patient survival. Lipin‐1 is critical for the survival of LUAD cells, but not for nontumorigenic human lung cells. Depletion of lipin‐1 activates ER stress response and causes dysregulation of cellular autophagy. Activation of IRE1*α*‐JNK pathway is required to ER stress and autophagy induction in lipin‐1‐deficient LUAD cells. In addition, the lack of lipin‐1 enhances the sensitivity of LUAD cells to cisplatin treatment. Our results suggest that lipin‐1 could be a potential target alone or combination with other treatment for lung cancer therapy.

## Materials and Methods

### Reagents

Cisplatin (P4394‐25MG) and JNK inhibitor (SP600125, #S5567) were purchased from Millipore Sigma (St. Louis, MO). Cisplatin was dissolved in DMSO at a concentration of 10 mmol/L, and aliquots were stored at −20°C. Stock solutions were diluted to the desired final concentrations with growth medium just before use. Antibodies against lipin‐1 (#14906), Akt (#4691), phospho‐Akt^Ser473^ (#4060), phospho‐Akt^T308^ (#13038), mTOR (#2972S), phospho‐mTOR^Ser2448^ (#2971), phospho‐mTOR^Ser2481^ (#2974), p70S6K (#9202), phospho‐p70S6K^Thr421/Ser424^ (#9204), phospho‐PKC*α*/*β* ^Thr638/641^ (#9375), phospho‐PKC*β* ^Ser660^ (#9375), phospho‐PKC*δ*/*θ* ^Ser643/676^ (#9376), phospho‐PKC*δ* ^Thr505^ (#9374), phospho‐PKC*θ* ^Thr538^ (#9377), phospho‐PKC*ζ*/*λ* ^Thr410/403^ (#9378), phospho‐PKD1^Ser916^ (#2051), phospho‐PKD1^Ser744/748^ (#2054), PKD1 (#2052), PDK3 (#5655), Beclin‐1 (#3495), SAPK/JNK (#3708), phosphor‐SAPK/JNK^Thr183/Tyr185^ (#4668), eIF2*α* (#5324), phospho‐eIF2*α* ^Ser51^ (#3398), BiP/GRP78 (#3177), caspase‐3 (#9665) and phospho‐ULK1 (#5869), IRE1*α* (#3294), CHOP (#2895), PERK (#5683), phospho‐PERK (#3179), and ATG5 (#2630) were purchased from Cell Signaling Technology (Danvers, MA). Phospho‐PKD2^Ser876^ (#07‐385), PKD2 (#07‐488), ATG7 (#MABN1124), LC3B (#L7543), p62 (P0067), and *β*‐actin (A1978) were purchased from Millipore Sigma. ATF6*α* (ab122897), phospho‐IRE1*α* (ab48187), XBP1 (ab37152), and ULK1 (ab128859) were ordered from Abcam (Boston, MA). LAMP‐1 antibody (sc‐20011) was purchased from Santa Cruz Biotechnology (Dallas, TX). Phospho‐Beclin‐1^T119^ antibody (#AP3765a) was purchased from Abgent Biotech (Suzhou, Jiangsu Province, China). HRP‐conjugated secondary antibodies were purchased from Thermo Fisher Scientific (Waltham, MA).

### Plasmids

The control firefly luciferase shRNA (sh*Luc*) and two *LPIN1*‐specific shRNAs (sh*LPIN1*#1 and sh*LPIN1*#2) were a kind gift from Dr. Guangwei Du 5. The target sequences for sh*Luc*, sh*LPIN1*#1, and sh*LPIN1*#2 are GATTTCGAGTCGTCTTAAT, GTGGTTGACATAGAAATCA, and GCAGAACTCTTCCTAATGA, respectively. The oligos of shRNA targeting *IRE1α* were synthesized in Genewiz (Suzhou, China) and cloned in pLKO.1 lentiviral vector. The target sequence is GCCCGGCCTCGGGATTTTT. The original GFP‐LC3 (#22405) and mRFP‐GFP‐LC3 (#22418) expression plasmids were ordered from Addgene 9. For lentivirus‐mediated expression, the cDNA fragment of GFP‐LC3 or mRFP‐GFP‐LC3 was cloned into pCDH‐CMV‐MCS‐EF1‐puro plasmid.

### Patients and specimens

The tumor samples from a total of 16 patients were used in this study. The patients did not receive any preoperative cancer treatment. Clinical samples were collected from these patients after obtaining informed consent according to an established protocol approved by the Ethics Committee of Quzhou People's Hospital.

### Lentivirus production and transduction

The delivery of expression constructs cells was through lentiviral infection. Viruses were generated in 293T cells. To produce virus, plasmids including the lentiviral shRNA vector, pCMVR8.74, and pMD2.G were cotransfected into 293T cells using LipofectAMINE Plus reagent from Life Technologies (Carlsbad, CA) according to the instruction. At 48 h post‐transfection, virus‐containing supernatants were collected and centrifuged at 3000 *g* for 5 min to remove suspended target cells. The supernatants were mixed with polybrene at final working concentration of 10 *μ*g/mL for infection. Medium containing virus was replaced with fresh growth medium 6 h after viral infection. The cells were used for experiments 2–3 days post‐transduction.

### Cell culture and viability measurement

HEK293T lentiviral packaging cell was obtained from Dr. Xiaolin Wu's laboratory. All cancer cell lines in this study were obtained from American Type Culture Collection (ATCC, Manassas, VA) and were cultured in Dulbecco's modified Eagle Medium (DMEM, Thermo Fisher Scientific) supplemented with heat inactivated 10% fetal bovine serum (FBS, Sigma‐Aldrich, St. Louis, MO). For viability measurement, cells were trypsinized and stained with trypan blue solution. Trypan blue‐negative viable cells and trypan blue‐positive dead cells were counted under a light microscopy.

### Cell apoptosis assay

LUAD cells transduced with lentiviruses were harvested and double stained with FITC Annexin‐V and PI (Biolegend, San Diego, CA) according to the manufacturer's instructions. The apoptotic cells were analyzed by flow cytometry (Beckman Coulter, Brea, CA).

### Colony formation assay

Cells were plated into six‐well culture plates covered by soft agar and cultured for 14 days to allow colony formation. The medium was changed every 3 days. The cells were fixed with 4% formaldehyde for 15 min and stained with 0.1% crystal violet for 15 min. Colonies were photographed, and the number of colonies was counted using ImageJ (National Institutes of Health, Bethesda, MD) from three independent experiments.

### Immunohistochemical and immunofluorescence staining

For immunohistochemical analysis, tissues were fixed overnight with 4% paraformaldehyde solution. Sections were prepared according to standard procedure. Paraffin‐embedded sections were deparaffinized with xylene, dehydrated in decreasing concentrations of ethanol, and then processed in 10 mmol/L citrate buffer (pH 6.0) and heated in microwave oven for 15 min for antigen retrieval. Tissue sections were treated with 3% hydrogen peroxidase in PBS for 10 min to block endogenous peroxidase activity. Sections were incubated with blocking serum for 30 min and incubated with lipin‐1 primary antibody over night at 4°C. The staining procedure was followed the manufacturer's instructions of ABC staining system (Santa‐Cruz Biotechnology, Santa Cruz, CA). Lipin‐1 IHC assessment was conducted as *Tomoyuki Mashimo* described 10. Briefly, the immunostained lung tissue slides were scored manually by assigning a value for staining intensity on a scale of 0–3 and a value representing the proportion of stained tumor cells or normal cells on a scale of 0–100%. These two values (intensity and percentage of positive cells) were then multiplied to obtain a histoscore (range 0–300), which was used for further analyses. For each sample, five slides of 400X fields were evaluated. The final count represented the mean of histoscore from these five slides.

For immunofluorescence staining, cells were cultured in EBSS for 24 h. The staining protocol was described previously 11. Briefly, cells were fixed with 4% paraformaldehyde and permeabilized with 0.1% Triton X‐100 in PBS. After blocking in PBS containing 5% normal goat serum for 30 min at room temperature, cells were stained with primary antibodies, followed by appropriate fluorescent dye‐conjugated secondary antibodies. Coverslips were mounted on to microslides with 4% propyl‐gallate mounting solution. All the immunofluorescent images were captured by a Nikon Confocal Laser Microscope (Minato, Tokyo, Japan).

### Real‐time PCR

Total RNA was extracted from cells by TRIzol Reagent (#DP424, Tiangen Biotech Co. Ltd, Beijing, China) according to the manufacturer's protocol and reverse‐transcribed using Maxima Reverse Transcriptase (#EP0742; Thermo Fisher Scientific). Real‐time PCR was performed in triplicate using SGExcel^R^ FastSYBR Mixture (#B532955; Sangon Biotech Co. Ltd, Shanghai, China) on Roche LightCyclerR 480 Quantitative PCR System (Indianapolis, IN). The following primers were used for the PCR: total lipin‐1 forward: 5′‐TGCTGGAGAGCAGCAGAACTC‐3′, reverse: 5′‐TAGGGTATGAGGCTGACTGAG‐3′; lipin‐1a forward: 5′‐TGCTGGAGAGCAGCAGAACTC‐3′, reverse: 5′‐CGGAAGGACTGGGAGTGGGT‐3′; lipin‐1b forward: 5′‐AGCCTCATACCCTAATTCGGAT‐3′, reverse: 5′‐TCCGAAGGATGGAACAGGGAAGA‐3′. Relative expression levels was normalized to *β*‐actin and calculated by 2‐ΔΔct method. Each experiment was performed independently at least three times.

### Western blotting

Cells were harvested 2–3 days postviral infection and lysed using 1X SDS lysis buffer containing protease inhibitor cocktail (Roche) and phosphatase inhibitor cocktail (1 mmol/L sodium orthovanadate, 5 mmol/L sodium fluoride, 3 mmol/L *β*‐glycerophosphate, and 4 mmol/L sodium tartrate). The lysates were centrifuged at 17,000 g for 20 min at 4°C, and the supernatants were collected. Identical amounts of protein extracts were separated by SDS‐PAGE and subjected to gel transfer. After blocking for 1 h at room temperature in a Tris‐buffered saline containing 0.1% Tween‐20 and 0.5% casein, membranes were incubated overnight at 4°C with primary antibodies and followed by incubation with HRP‐conjugated secondary antibodies for an hour at room temperature. Immune complexes were detected by the Tanon 4200SF system from Tanon Biotechnology (Shanghai, China). Band intensity was quantified using the program supplied by the manufacturer.

### Bioinformatic analysis

We evaluated the correlation of lipin‐1 expression levels to patient survival in lung cancer using the KMplotter (http://kmplot.com/analysis) 12. Patients were split by median lipin‐1 expression, and analysis was performed the lung adenocarcinoma dataset irrespective of grade, stage, or prior treatment regimen.

### Statistical analysis

All statistical analyses were performed using Prism software (GraphPad Software, La Jolla, CA). Values reported are expressed as the mean ± SD. Student's *t*‐test and ANOVA were used for statistical analysis, as applicable. Statistical significance was set at *P* < 0.05.

## Results

### Lipin‐1 is overexpressed in lung adenocarcinoma and its overexpression associates with poor prognosis

**Figure 1:**
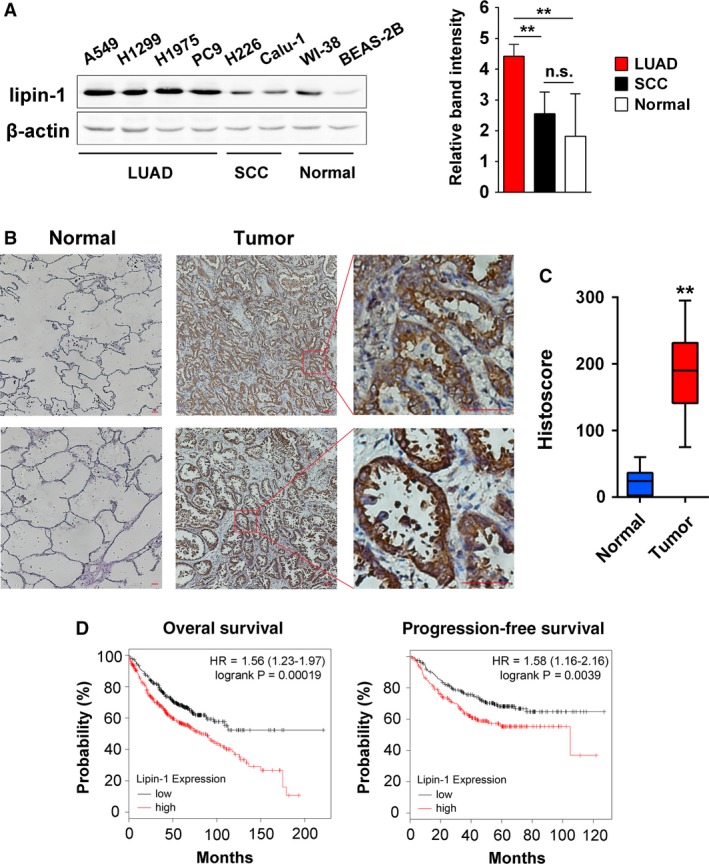
Lipin‐1 is overexpressed in lung adenocarcinoma and is correlated with poor prognosis. (A) Lipin‐1 protein expression in lung cell lines. Left, representative result of three independent experiments was shown. Right, classification and quantification of band intensity on the left. The intensity of bands was normalized by *β*‐actin expression. (B) Representative images of lipin‐1 expression in normal lung tissue and lung adenocarcinoma samples are shown. Scale bars, 200 *μ*m (C) The histoscore of lipin‐1 expression for (B). Sixteen NSCLC patient samples and 10 normal lung tissue samples were included. Histoscore was calculated by multiplying the staining intensity value and the percentage of positive cells. (D) Correlation of lipin‐1 expression with patient survival in lung adenocarcinoma. Probability of overall survival and progression‐free survival with lung adenocarcinoma stratified on low (black) versus high (red) expression levels of lipin‐1 was obtained from Kaplan–Meier Plotter/lung cancer (http://www.kmplot.com/lung). **P < 0.01; n.s., no significance.

At first, we examined lipin‐1 expression in various lung cell lines including lung adenocarcinoma (A549, NCI‐H1299, NCI‐H1975, and PC‐9), squamous cell carcinoma (NCI‐H226, Calu‐1),and noncancerous lung cell lines (WI‐38, BEAS‐2B). The protein level of Lipin‐1 was significantly upregulated in lung adenocarcinoma cells, while it also expressed in both squamous cells and normal lung cells in lower level (Fig. 1A). Lipin‐1 has two major isoforms named lipin‐1a and lipin‐1b. It has been reported that lipin‐1a is prone to localize in nucleus while lipin‐1b stays in cytoplasm under normal condition 13. We examined lipin‐1 mRNA expression using lipin‐1a and lipin‐1b specific primers. Lipin‐1b mRNA was markedly elevated in LUAD but not in SCC cell lines. The change of lipin‐1a mRNA expression was not significant, suggesting lipin‐1b made major contribution to lipin‐1 upregulation in LUAD cell lines (Fig. S1). Next, we determined the expression of lipin‐1 in tissue samples. Compared to normal lung tissue, lung adenocarcinoma tumor samples showed much higher expression of lipin‐1, which was dominantly localized in cytosol of tumor cells (Fig. 1B). Histoscore was calculated to evaluate overall difference of staining intensity and the proportion of positive cells between adenocarcinoma and normal lung tissue (Fig. 1C). Importantly, analysis of correlation between lipin‐1 expression and prognosis from microarray database revealed that lipin‐1 overexpression was strongly associated with poor prognosis in patients with lung adenocarcinoma (Fig. 1D).

### Lipin‐1 is required for the survival of lung adenocarcinoma cells

**Figure 2:**
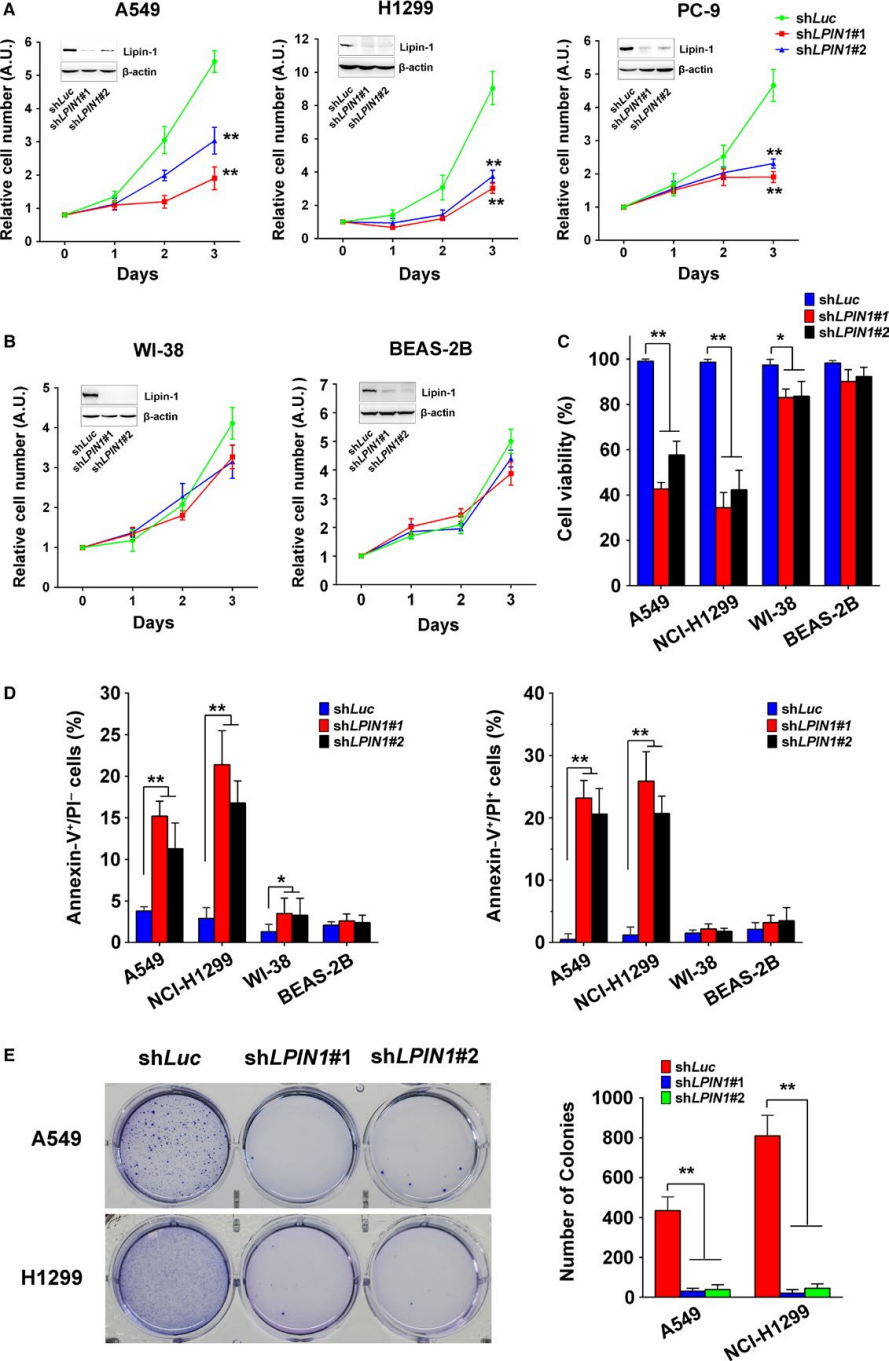
Lipin‐1 is required for the survival of lung adenocarcinoma cells. (A) Lipin‐1 knockdown decreased lung adenocarcinoma cell proliferation. Cells were counted daily three days after infected with lentiviruses carrying the control or *LPIN1* shRNAs. *N* = 3; **P < 0.01. (B) Lipin‐1 knockdown had little effect on nontumorigenic lung cell lines. *N* = 3. (C) Lipin‐1 knockdown reduced cell viability in A549 and H1299 cells. Viable and dead cells were distinguished by trypan blue exclusion test. Cells were collected and stained by trypan blue solution 3 days after lentiviral transduction. *N* = 3; *P < 0.05; **P < 0.01. (D) Cells were stained by FITC‐conjugated Annexin‐V and PI and tested by flow cytometry. Early apoptotic (Annexin‐V^+^/PI ^−^) and late apoptotic/necrotic (Annexin‐V^+^/PI ^+^) cells were calculated, respectively. *N* = 3; *P < 0.05; **P < 0.01. (E) Lipin‐1 deficiency inhibited colony formation in soft agar. Cells were seeded to soft agar with puromycin after lentivirus transduction and cultured for 3 weeks. Number of colonies was counted by software, and representative images are shown in left panel. *N* = 3; **P < 0.01.

The studies on lipin‐1 have been mainly focused on metabolic dysregulation, such as lipodystrophy 14, obesity, hypertriglyceridemia 15, rhabdomyolysis, and myopathy 16, 17. Although it was reported recently that lipin‐1 is crucial to breast and prostate cancer cells 4, 5, it remains unknown whether lipin‐1 also contributes to cell survival of lung cancer cells. We knocked down lipin‐1 through lentivirus‐mediated shRNA in A549, NCI‐H1299, and PC‐9 adenocarcinoma cells. Both shRNAs targeting lipin‐1 showed significant effect on the number of viable cells (Fig. 2A). Previous study revealed nontumorigenic human mammary epithelial cells (HMECs) and estrogen receptor‐positive breast cancer cells were less sensitive to lipin‐1 knockdown compared with TNBC cells 5. Herein, we examined whether lipin‐1 knockdown had similar specificity in lung cancer. The depletion of lipin‐1 had much less impact on the number of viable WI‐38 and BEAS‐2B cells (Fig. 2B). Trypan blue staining showed that lipin‐1 deficiency dramatically decreased cell viability of A549 and H1299 cells, implying reduced cell number may be at least partially a result of increased cell death (Fig. 2C). In addition, we analyzed the type of cell death in lipin‐1‐depleted condition by flow cytometry. FITC‐conjugated Annexin‐V and PI double‐staining assay showed lipin‐1 knockdown increased both cell apoptosis (Annexin‐V^+^/PI^−^) and necrosis (Annexin‐V^+^/PI^+^) in LUAD cells (Fig. 2D). Next, we performed soft agar assay to assess lipin‐1 depletion on tumorigenic ability of LUAD cells. The result showed lack of lipin‐1 essentially impaired the colony formation of A549 and H1299 cells, suggesting lipin‐1 is indispensable for the progression of lung adenocarcinoma (Fig. 2E).

### Lipin‐1 depletion disrupts intracellular autophagy homeostasis

**Figure 3:**
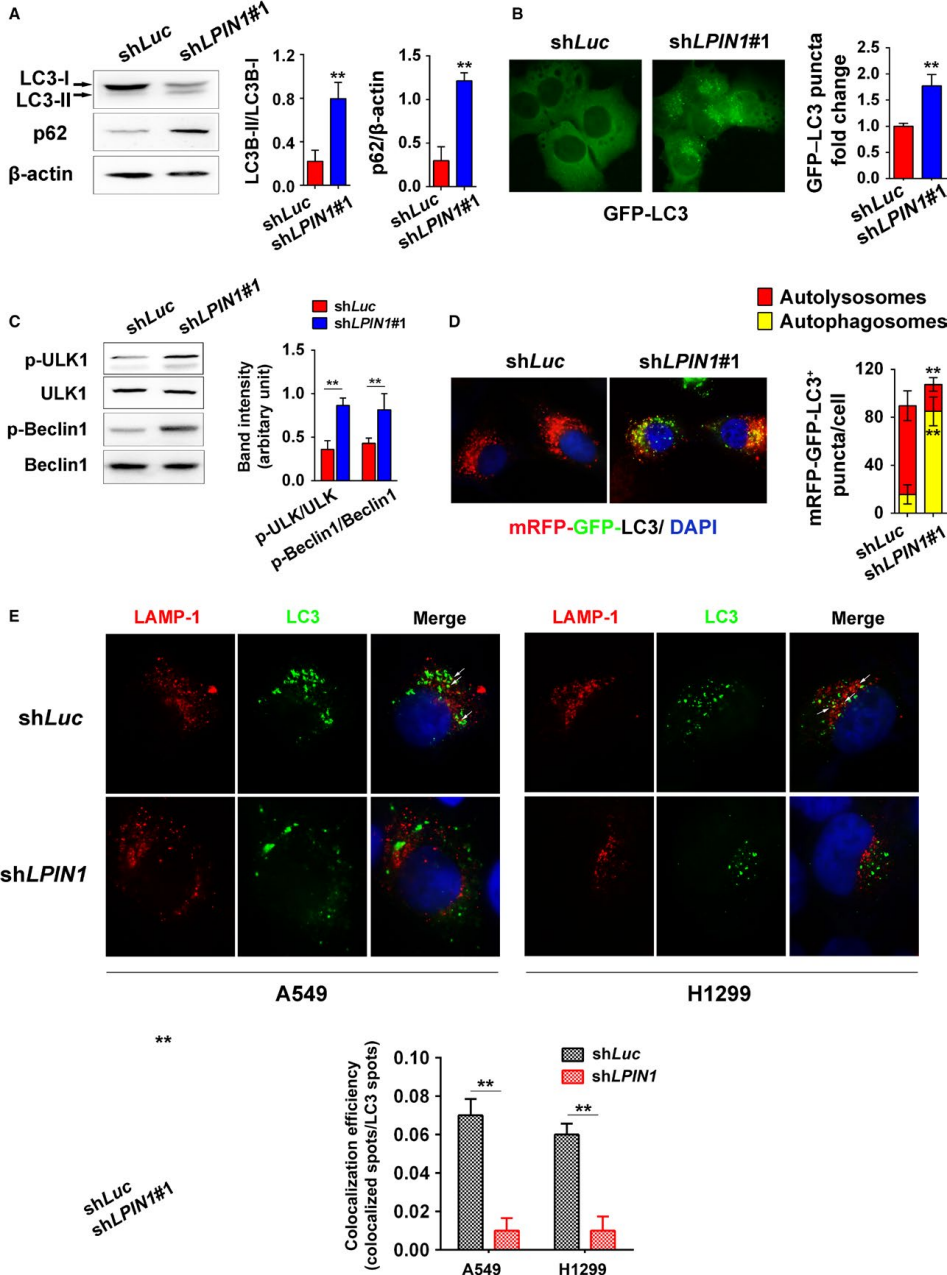
Lipin‐1 depletion disrupts intracellular autophagy homeostasis. (A) Left, A549 cells were collected and lysed 36 h after transduction with sh*Luc* or sh*LPIN1*#1. LC3 and p62 proteins were examined by Western blot. Right, the intensity of bands was calculated by ImageJ. *N* = 3; **P < 0.01. (B) A549 cells expressing GFP‐LC3 were fixed. Images were taken under 100× magnification oil objective lens of fluorescence microscope. GFP‐LC3 puncta in the cells of 10 fields were counted, and relative fold change was calculated. **P < 0.01. Left, representative images. (C) Cell lysate of A549 was subjected to Western blot to determine the status of ULK and Beclin‐1. Right, the intensity of the left bands, normalized by total protein levels. *N* = 3; **, *P* < 0.01. (D) Left, autophagosome (yellow puncta) or autolysosome (red puncta) formation in A549 cells. Cells expressing mRFP‐GFP‐LC3 were cultured in EBSS for 3 h. Right, quantification of mRFP‐GFP‐LC3 puncta (100 cells per group). **P < 0.01. (E) Cells were cultured in EBSS for 6 h. Colocalization efficiency of LAMP‐1 (red) and endogenous LC3 (green) in A549 and H1299 cells were captured by confocal microscopy and counted (lower). For each treatment, the events in at least 20 cells were included. **P < 0.01.

Lipin‐1 deficiency was reported to change intracellular autophagy in noncancerous and cancer cells 5, 17. Herein, we assessed autophagy in LUAD cells after lipin‐1 depletion by measuring the change of LC3 and p62 proteins. LC3‐II, the phosphatidylethanolamine‐conjugated form of LC3‐I which is an marker to estimate the induction of autophagy, was increased in lipin‐1‐deficient A549 cells (Fig. 3A). Cells expressing GFP‐LC3 fusion protein also showed much more GFP puncta under lipin‐1 knockdown condition (Fig. 3B). We also detected the alteration of other crucial components for the machinery of autophagy. The phosphorylation of ULK and Beclin‐1 was obviously increased after lipin‐1 knockdown, while the amount of ATG5 and ATG7 was not significantly changed (Fig. 3C; Fig. S2). These results suggested autophagy initiation was enhanced under lipin‐1‐deficient condition. However, as an indicator reflecting the fusion of autophagosome‐lysosome and clearance of autophagy, p62 was higher in the cells transduced with lipin‐1 shRNA, implying silencing of lipin‐1 might disrupt the sequestration of autophagy (Fig. 3A). In addition, we evaluated the autophagosome and autolysosome formations by expressing mRFP‐GFP‐LC3 in A549 cells. There were much more yellow puncta (autophagosomes) in A549 cells lack of lipin‐1 compared with WT cells under nutrient deprivation condition. Meanwhile, the red puncta (autolysosomes) were obviously less in lipin‐1‐depleted cells, indicating impaired maturation of autophagosome to form autolysosome (Fig. 3D). To further explore the underlying mechanism of defect autophagy clearance, the fusion of autophagosomes with lysosomes was examined in LUAD cells. Nutrient deprivation induced the fusion of autophagosomes with lysosomes to form autolysosome indicated by the perinuclear colocalization of LC3 and lysosome marker LAMP‐1 (Fig. 3E). By contrast, lipin‐1 depletion significantly reduced the fusion between autophagosomes and lysosomes upon starvation in both A549 and H1299 cells (Fig. 3E). Taken together, lipin‐1 deficiency promoted intracellular autophagy initiation but disrupted autophagic flux by impeding the fusion of autophagosomes and lysosomes.

### Lipin‐1 deficiency activates autophagy through mTOR and PKC pathway

**Figure 4:**
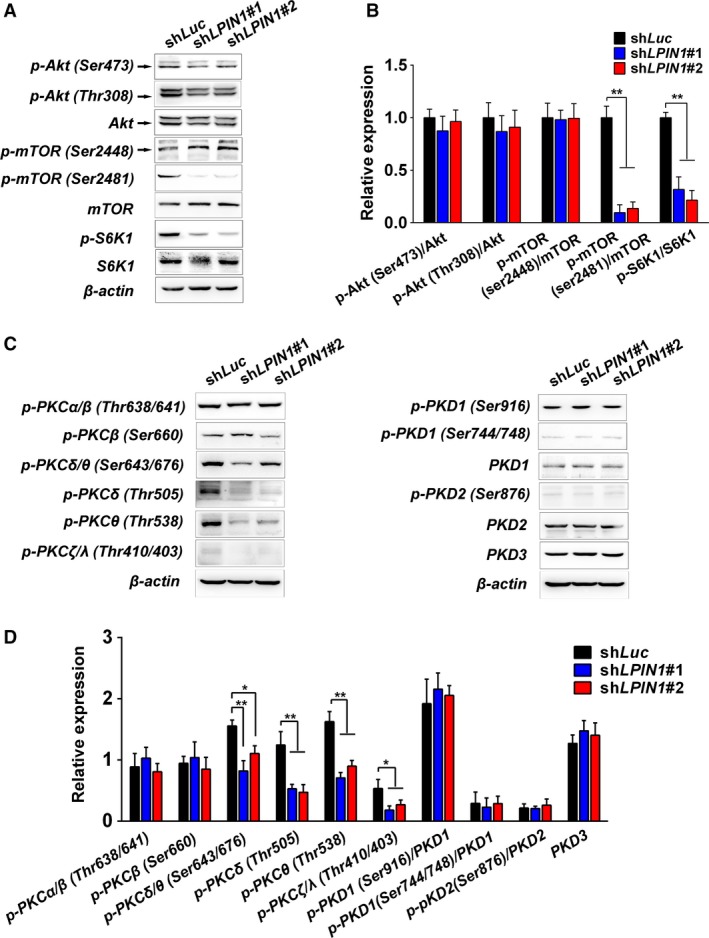
Lipin‐1 deficiency activates autophagy through mTOR and PKC pathway. (A) Cell lysate of A549 was subjected to Western blot to determine the activation of mTOR pathway. (B) The intensity of bands from (A). Values were normalized by the value of sh*Luc* in each antibody group. *N* = 3; *P < 0.05; **P < 0.01. (C) Western blot for the activation of PKC family in A549 cells. (D) The intensity of bands from (C). *N* = 3; *P < 0.05; **P < 0.01.

PI3K/Akt/mTOR pathway is the major regulator of cellular autophagy 18. We examined the activation of Akt and mTOR proteins. Akt activation seemed not to be influenced by lipin‐1 status (Fig. 4A). However, the phosphorylation of mTOR in Ser^2481^ was obvious suppressed in lipin‐1 knockdown cells. Concomitantly, the phosphorylation of S6K1, a hallmark of mTOR activation and correlated with autophagy inhibition, was decreased in lipin‐1‐deficient cells (Fig. 4A and B). Protein kinase C (PKC) pathway, including PKD, is involved in autophagosome formation and maturation 19, 20. Lipin‐1 knockout greatly attenuated the activation of PKD in mouse myocytes 17. We determined the activation of major components in PKC pathway by Western blot. Although the phosphorylation of classical members of PKC family, PKC*α* and PKC*β*, was not affected by lipin‐1 knockdown, the phosphorylation of nonclassical PKCs (PKC*δ*, PKC*θ*, PKC*ζ*/*λ*) was significantly reduced (Fig. 4B and D). Unlike the situation of myocytes, the inhibition of PKD1 phosphorylation in lipin‐1‐depleted LUAD cells was not prominent (Fig. 4D).

### Lipin‐1 knockdown triggered ER stress and is essential to autophagy induction and cell survival

**Figure 5:**
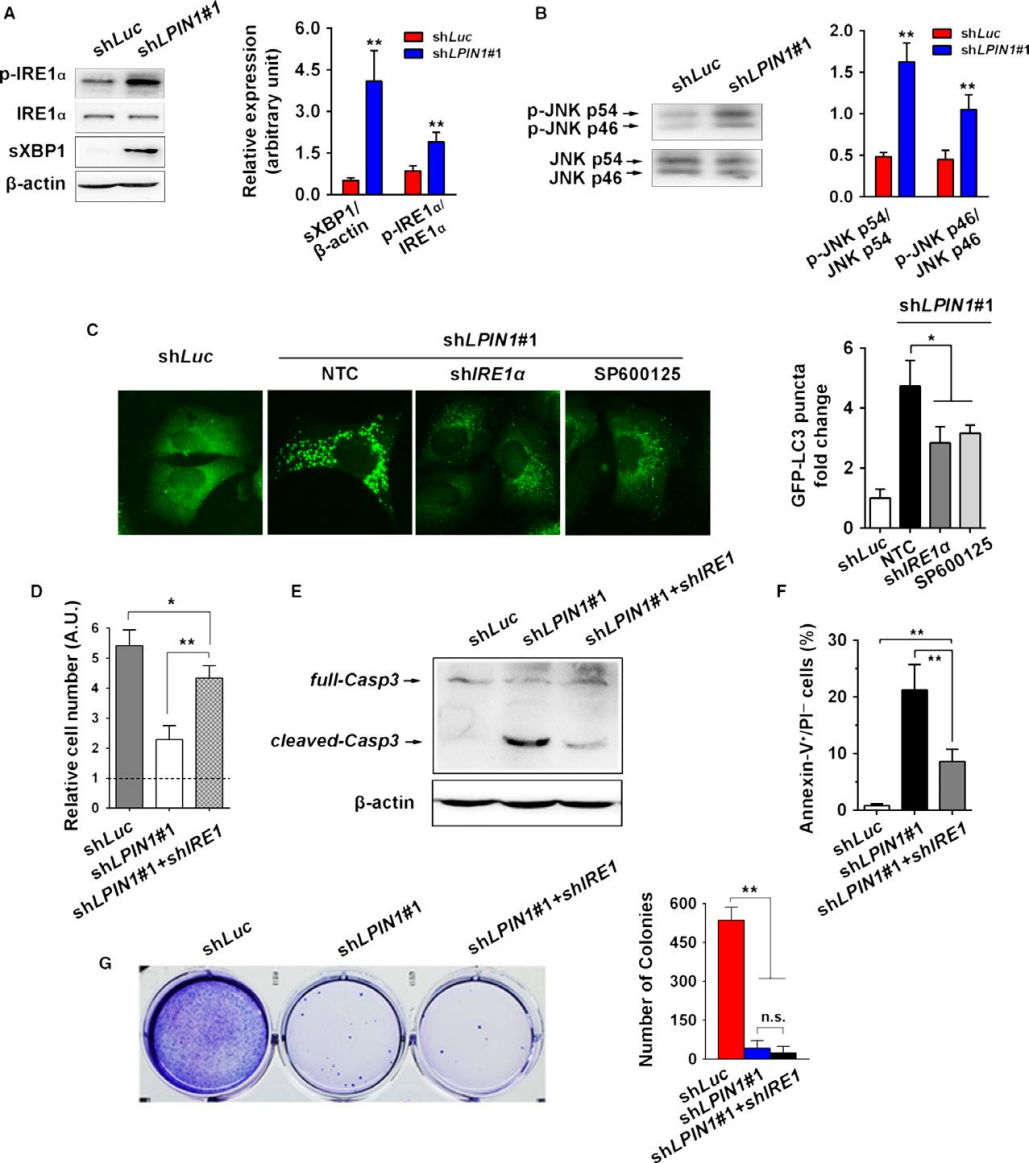
Lipin‐1 knockdown triggered ER stress and is essential to autophagy induction and cell survival. (A) Lipin‐1 knockdown led to a specific elevation of p‐IRE1*α* and spliced XBP1 (sXBP1) in A549 cells. Right, quantification of relative levels of the p‐IRE1*α* and sXBP1. p‐IRE1*α* and sXBP1 levels were normalized by total IRE1*α* and *β*‐actin, respectively (*N* = 3). (B) JNK phosphorylation level was increased in A549 cells expressing sh*LPIN1*. Right, quantification of the relative phosphorylation levels of phospho‐JNK p46 and phospho‐JNK p54. The levels of phospho‐JNK isoforms were normalized by their respective total JNKs (*N* = 3). (C) Inhibition of IRE1*α* by shRNA or suppression of JNK activity by SP600125 (40 nmol/L) reduced cellular autophagic flux. A549 cells expressing GFP‐LC3 were treated with indicated shRNAs or inhibitor. GFP‐LC3 puncta were counted as described previously. Right, representative images were shown. (D) Expression of sh*IRE1α* partially rescued cell proliferation decrease in lipin‐1 knockdown A549 cells. Cells were counted 3 days after lentivirus transduction. Expression of sh*IRE1α* reduced cell apoptosis in lipin‐1 knockdown cells indicated by cleaved caspase‐3 expression (E) and Annexin‐V/PI staining (F) (*N* = 3). (G) Inhibition of sh*IRE1α* did not recover the capability of colony formation impaired by lipin‐1 deficiency (*N* = 3). *P < 0.05; **P < 0.01; n.s., no significance.

Lipin‐1‐mediated phospholipid synthesis is important to membrane biogenesis, and its inhibition could lead to ER stress response in breast cancer cells. Similar to the situation in breast cancer cells 5, we found IRE1*α* branch was activated in lipin‐1‐deficient LUAD cells, indicated by increased phosphorylated IRE1*α* level, and dramatically upregulated sXBP1 (the spliced form of X‐box‐binding protein‐1; Fig. 5A), while the other indicators of ER stress were not changed (Fig. S3A). Activation of JNK has been reported in response to IRE1*α*‐dependent ER stress 21 and is involved in autophagy induction during ER stress 8. Our result showed that the phosphorylation of JNK was significantly enhanced after lipin‐1 knockdown (Fig. 5B). To confirm the effect of IRE1*α*‐JNK in autophagy regulation in lipin‐1‐deficient context, we inhibited IRE1*α* and JNK through shRNA‐mediated knockdown and JNK inhibitor SP600125, respectively. The GFP‐LC3 puncta resulted from lipin‐1 depletion were decreased after IRE1*α*‐JNK pathway was inhibited (Fig. 5C). Concomitantly, the cell phenotype was rescued in part in lipin‐1‐deficient cells after inhibition of IRE1*α*. Cell proliferation was significantly elevated even though it was still lower than sh*Luc* control cells (Fig. 5D). Meanwhile, the apoptosis of A549 reflected by caspase‐3 cleavage and Annexin‐V/PI staining was attenuated (Fig. 5E and F), and total cell death measured by trypan blue staining was reduced significantly (Fig. S3B). Nonetheless, repression of IRE1*α* did not rescue the deficiency of tumorigenic potential due to lack of lipin‐1 (Fig. 5G).

### Inhibition of lipin‐1 activity enhanced cisplatin sensitivity of LUAD cells

**Figure 6:**
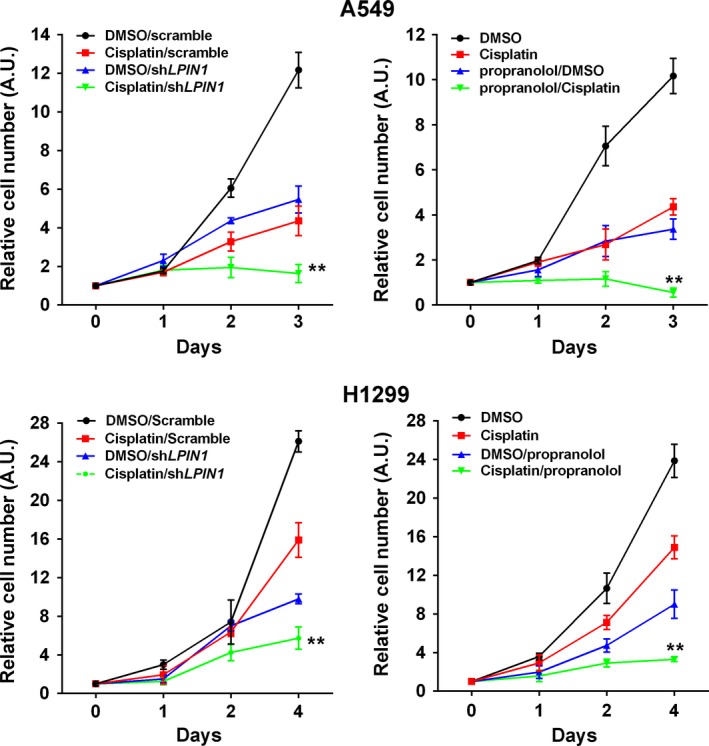
Inhibition of lipin‐1 activity enhanced cisplatin sensitivity of LUAD cells. A549 and NCI‐H1299 cells were treated either by sh*LPIN1* or propranolol (100 *μ*mol/L) alone or association with cisplatin (100 *μ*mol/L). *N* = 3, **P < 0.01, compared to cisplatin treatment group.

To determine the role of lipin‐1in current lung cancer therapy, we combined the inhibition of lipin‐1 activity and cisplatin treatment in LUAD cells. Inhibition of lipin‐1 activity was achieved by either shRNA or propranolol, which was documented as a lipin‐1 antagonist 4. Lipin‐1 inhibition with both shRNA and propranolol significantly enhanced the effect of cisplatin to A549 and NCI‐H1299 cells (Fig. 6).

## Discussion

In the present study, we analyzed lipin‐1 expression in patients with lung cancer. Lipin‐1 is significantly upregulated in lung adenocarcinoma (Fig. 1B). Correspondingly, high lipin‐1 expression is associated with poor prognosis of patients with LUAD (Fig. 1D). Knockdown of lipin‐1 by shRNAs in LUAD cells resulted in reduced cell viability and increased apoptosis (Fig. 2). Normal lung fibroblast WI‐38 and human bronchial epithelial cell BEAS‐2B were less sensitive to lipin‐1 depletion. Inhibition of lipin‐1 activity enhanced the sensitivity of cisplatin to lung adenocarcinoma cells (Fig. 6). Our current study demonstrated the crucial role of lipin‐1 in lung adenocarcinoma and the underlying mechanism, suggesting the intervention of lipin‐1 alone or combined with chemotherapeutic agents may provide new therapeutic strategies in treating patients with lung adenocarcinoma.

Lipin‐1 knockdown led to disturbance of intracellular homeostasis, including abnormal autophagy (Fig. 3) and ER stress (Fig. 5). DAG is the most important precursor for phospholipid synthesis. As a key enzyme for intracellular DAG production, the lack of lipin‐1 may result in imbalance of phospholipids on the ER membrane, especially in highly proliferative cancer cells. It could change ER structure or protein synthesis in the ER. In addition, the activation of XBP1 was reported to elevate the expression and activity of PC/phospholipid‐biosynthesized enzymes, increase levels of membrane phospholipids, and expand surface area and volume of rough ER 5. IRE1*α*‐XBP1 activation induced by lipin‐1 deficiency could be considered as self‐protective response to keep intracellular homeostasis in cancer cells. Furthermore, our results suggest that ER stress could be causative of elevated autophagy initiation, as inhibition of ER stress through shRNA‐mediated IRE1*α* knockdown and its downstream factor JNK by chemical agent obviously reduced autophagy in lipin‐1‐depleted context. Notably, inhibition of ER stress just partially rescued cell phenotype lipin‐1‐depleted condition, implying that autophagy disturbance might contribute to the phenotype as well. Despite suppression of ER stress decreased cell death and recovered cell proliferation in short period, it failed to rescue tumorigenic ability of lipin‐1‐deficient LUAD cells (Fig. 5G), emphasizing the indispensable role of lipin‐1 in the progression of lung adenocarcinoma.

As a central regulator of cell growth, mTOR plays an important role at the interface of the pathways that coordinate the balance between cell growth and autophagy in response to nutritional status, growth factor, and stress signals 22. Inactivation of mTORC1 induces autophagy under various cellular stresses 23. Akt has been recognized as a major regulator of mTORC1 activity. In our study, mTORC1 phosphorylation was decreased while Akt phosphorylation was not significantly changed in lipin‐1 knockdown cells (Fig. 4A and B), suggesting lipin‐1 affects mTOR activation is independent of Akt activity. It has been reported mTORC1 regulates lipin‐1 subcellular localization by its kinase activity to control SREBP pathway 24. Further study is needed to demonstrate how lipin‐1 modulates mTOR activity in cancer cells.

Diacylglycerol, a lipid second messenger, was reported to be required for clearance of bacteria by autophagy in mammalian cells 25. DAG activates a protein kinase C (PKC) signaling cascade to regulate autophagosome formation and maturation 17, 19, 20. The requirement for lipin‐1 in autophagy clearance in myocytes raised the possibility that the production of DAG by lipin‐1 is important in this process 17. Unlike the situation in myocyte, the lack of lipin‐1 repressed the phosphorylation of several PKCs rather than PKD, revealing the effect of lipin‐1 deficiency on the activity of PKC family is context dependent. The elaborate relation of lipin‐1 and individual member of PKCs is attractive to be further explored.

In this study, we used propranolol as lipin‐1 antagonist to pharmacologically inhibit lipin‐1 activity. Although propranolol has long been known to be an inhibitor of PAP‐1^26^, it is not a lipin‐1‐specific antagonist. It also represses AKT and S6 protein phosphorylation 4. To exclude the possibility that the synergistic effect of cisplatin and propranolol treatment was mainly due to other off targeted effect rather than lipin‐1 inhibition, we inhibited lipin‐1 activity by its specific shRNA. Lipin‐1 inhibition by both shRNA and propranolol showed similar synergistic effect with cisplatin in A549 and H1299 cells, suggesting this effect was resulted from lipin‐1 dysfunction.

It is known that autophagy is commonly induced in the process of cancer chemotherapy. Generally, autophagy is considered to be a reason of chemotherapy resistance 27, 28. In our study, lipin‐1 knockdown induces the initiation of autophagy in LUAD cells (Fig. 3A). Meanwhile, lipin‐1 activity inhibition seems to enhance the sensitivity of LUAD cells to cisplatin treatment (Fig. 6). We suppose that the apparently contradictory could be explained through the imbalanced intracellular homeostasis resulted from disrupted autophagy when lipin‐1 was silenced. Lipin‐1 knockdown increases autophagy initiation and p62 expression simultaneously from Western blot examination (Fig. 3). p62 is an ubiquitin‐binding protein that plays an important role in autophagy. Protein aggregates formed by p62 can be degraded by the autophagosome. p62 binds to autophagosomal membrane protein LC3, bringing p62‐containing protein aggregates to the autophagosome. Lysosomal degradation of autophagosomes leads to a decrease in p62 levels during autophagy; conversely, autophagy inhibitors stabilize p62 levels 29, 30. In our study, lipin‐1 depletion caused enhanced autophagy initiation indicated by increased GFP‐linked LC3 dots and ULK phosphorylation (Fig. 3A–C), while the maturation of autolysosome was blocked (Fig. 3D and E). Considering previous reports of lipin‐1 and autophagy in myocytes 17, we think that both lipin‐1 deficiency and cisplatin treatment trigger intracellular autophagy, while the degradation of the autophagic substrates is disrupted in lipin‐1‐knockdown condition. The disturbance of intracellular homeostasis may explain why combination lipin‐1 inhibition and cisplatin treatment could strengthen the effect of cisplatin's cytotoxicity.

## Conflict of Interest

The authors have no conflict of interest.

## Supporting information


**Figure S1.** The mRNA expression of different Lipin‐1 isoforms.

**Figure S2.** The expression of other key components for autophagy complex machinery.

**Figure S3.** Lipin‐1 knockdown triggered ER stress through IRE1α branch.
