# Supplementary figures and images for: Lipin‐1 determines lung cancer cell survival and chemotherapy sensitivity by regulation of endoplasmic reticulum homeostasis and autophagy
Source: Cancer Med. 2018 Apr 16;7(6):2541–54. doi: 10.1002/cam4.1483 (PMC6010863; doi:10.1002/cam4.1483)

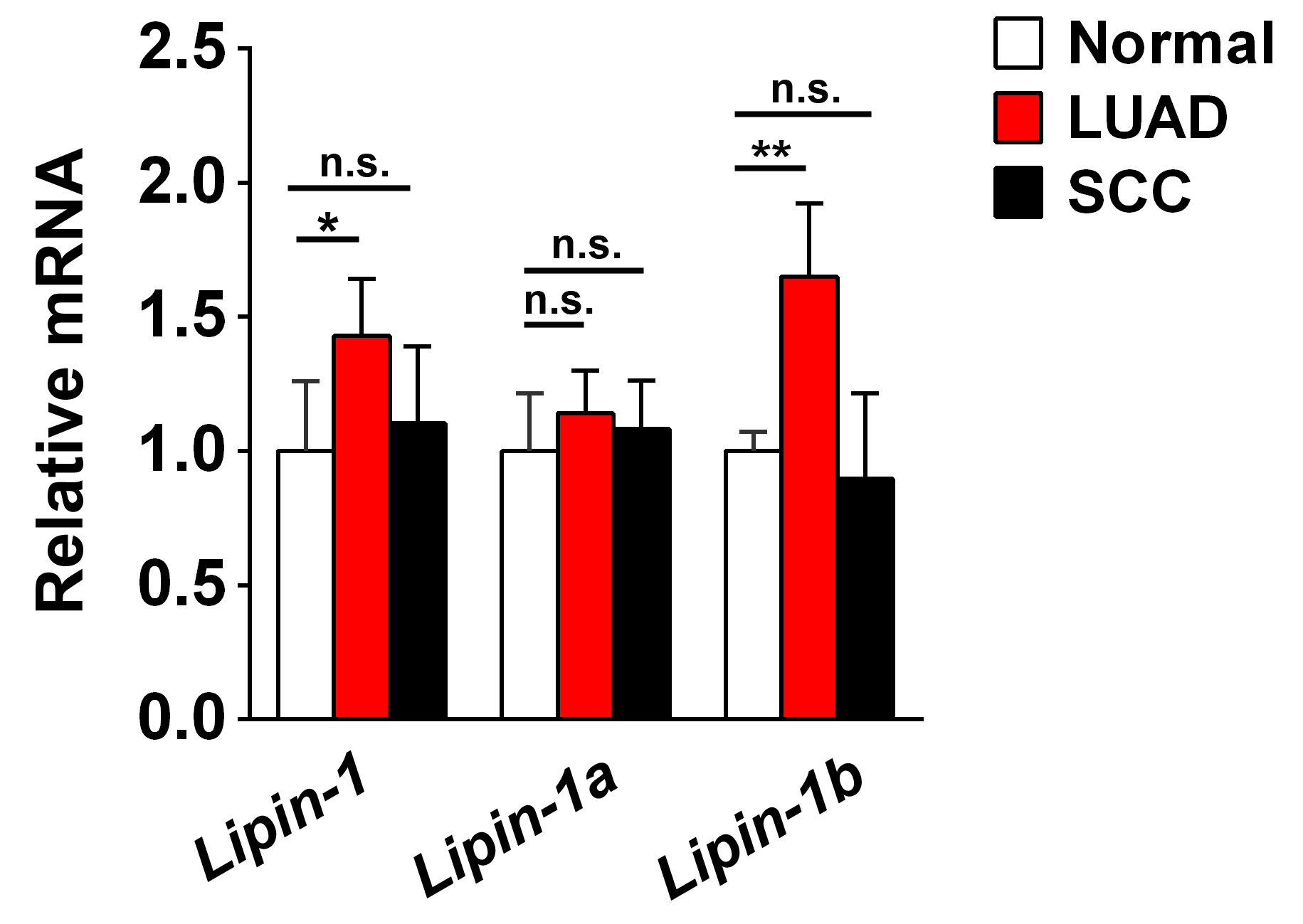

Supplement: Supplementary file 1 — Figure S1. The mRNA expression of different Lipin‐1 isoforms. [file CAM4-7-2541-s001.tif]

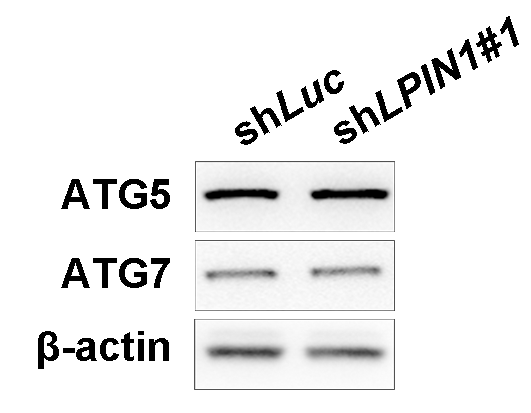

Supplement: Supplementary file 2 — Figure S2. The expression of other key components for autophagy complex machinery. [file CAM4-7-2541-s002.tif]

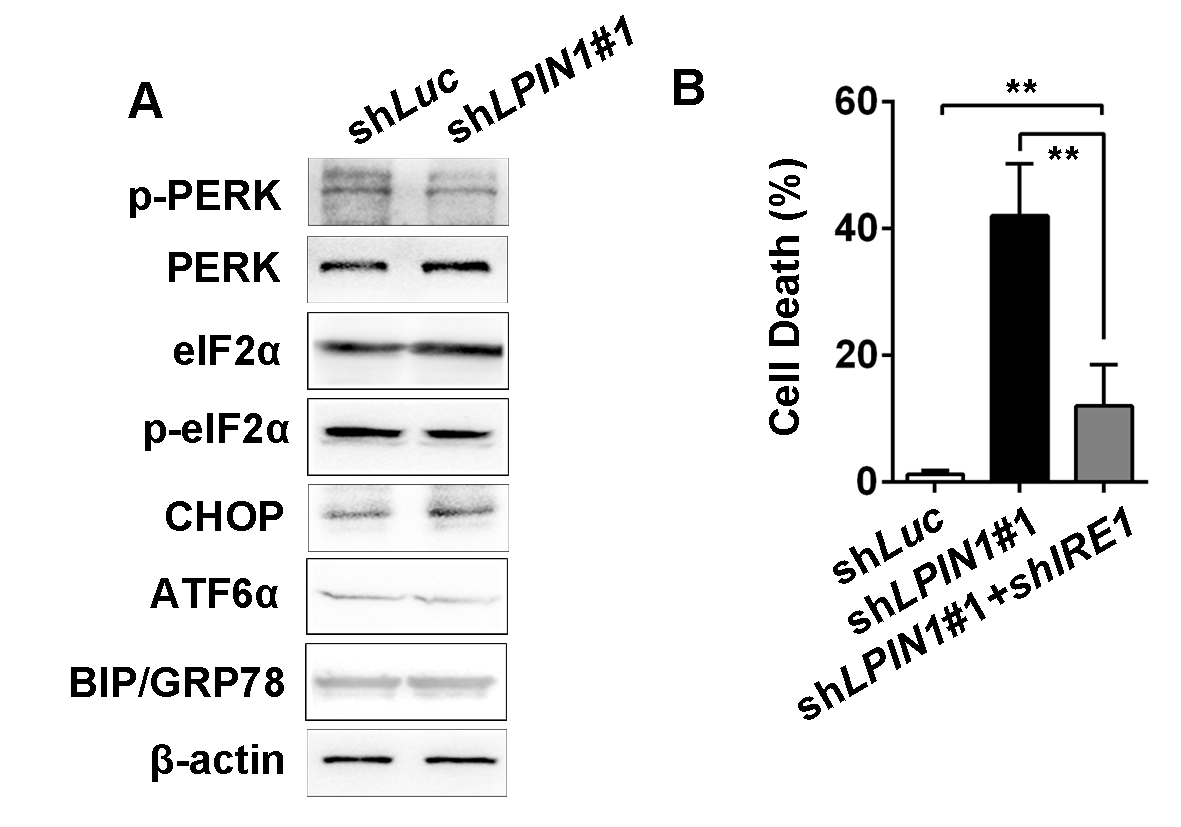

Supplement: Supplementary file 3 — Figure S3. Lipin‐1 knockdown triggered ER stress through IRE1α branch. [file CAM4-7-2541-s003.tif]
